# Bile Acid Conjugates with Anticancer Activity: Most Recent Research

**DOI:** 10.3390/molecules26010025

**Published:** 2020-12-23

**Authors:** Maria Luisa Navacchia, Elena Marchesi, Daniela Perrone

**Affiliations:** 1 Institute of Organic Synthesis and Photoreactivity, National Research Council, Piero Gobetti 101, 40129 Bologna, Italy; 2 Department of Chemical and Pharmaceutical Sciences, University of Ferrara, Luigi Borsari 46, 44121 Ferrara, Italy; mrclne@unife.it

**Keywords:** bile acids conjugates, natural product hybrids, anticancer agents, pharmacological activity, bioconjugation, drug design

## Abstract

The advantages of a treatment modality that combines two or more therapeutic agents in cancer therapy encourages the study of hybrid functional compounds for pharmacological applications. In light of this, we reviewed recent works on hybrid molecules based on bile acids. Due to their biological properties, as well as their different chemical/biochemical reactive moieties, bile acids can be considered very interesting starting molecules for conjugation with natural or synthetic bioactive molecules.

## 1. Introduction

Cancer is a multifactorial disease including interactions of complex genetic and environmental factors. More access to high-quality health care has led to increased cancer survival; furthermore, prevention has reduced deaths for some cancers (i.e., colorectal, female breast and prostate cancer) in the last decade. Nevertheless, cancer is the second leading cause of death globally and the WHO estimated that 9.6 million people worldwide died from cancer in 2018.

Conventional anticancer chemotherapies are frequently associated with significant levels of toxicity and/or multidrug resistance; additionally, many advanced and metastasized cancers remain untreatable. Therefore, there is a strong need to discover more effective and selective novel anticancer drugs. Countless natural products isolated from living organisms (i.e., microbes and plants) have been shown to possess pharmacological activities such as antimicrobial, anti-inflammatory, antiviral and anticancer. Despite the fact that the development of drugs based on bioactive natural products remains challenging, mainly because of difficulties associate with isolation, synthesis, mechanistic understanding and so on, several cytotoxic natural products have been developed as clinical drugs. For instance, taxanes, originally derived from *Taxus brevifoglia* [1], and vinca alkaloids from *Catharanthus roseus* [2], have been used to treat many types of advanced and/or metastatic cancers. Thus, synthetic efforts to novel compounds based on natural products have been primarly addressed to improve pharmacological features in the same biological space. Nevertheless, natural products have become one of the major sources of components in hybrid molecules due to their high potential, variety and pronounced biological activities. Among the extremely numerous bioactive natural products, there are classes of compounds containing potential reactive centers that enable the formation of covalent bonds, for instance, with amino acid hotspots on proteins or with other biological molecules, to modulate their biological action or with chemical species to yield hybrid drugs. The latter category represents an interesting avenue for the development of new, natural hybrid drugs. In 2003 Tietze, et al. [3] reviewed natural product hybrids in drug discovery, arriving at the conclusion that bioconjugation was a promising approach for the development of new lead compounds for medicine. Nowadays, the design and synthesis of natural-based hybrid compounds as multitargeted anticancer agents is a research field in great expansion. Hybrid drugs can provide combination therapies in a single multifunctional agent, resulting more specific and powerful treatments than conventional classic therapies which might also reduce side effects and the development of drug-resistance. With respect to combinatorial chemistry, the development of natural hybrid drugs offers the advantage of high structural diversity and biodiversity, and the intrinsic biological activity of the natural components of hybrids. The design of hybrid molecules represents an interesting approach to enhance biological activity with respect to both activity and selectivity, and to reduce potential drawbacks with respect to the hybrid components alone. Hybrids can be more than the sum of their components; they can also act synergistically and improve upon their simple drug combinations [4].

**Figure 1:**
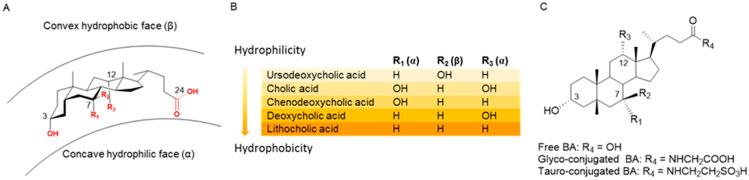
Structure and hydrophobicity of bile acids. (**A**) Perspective structure of BA: the number and the disposition of OH groups affect their hydrophobicity. (**B**) general hydrophobicity scale of BAs. (**C**) chemical structure of free and conjugated BAs.

This review focuses on the design and synthesis of hybrid products incorporating two distinct entities in one molecule where at least one moiety, the bile acid, is a bioactive natural product. Over the years, our research group has paid particular attention to bile acid (BA), i.e., endogenous natural molecules which are considered very interesting scaffolds due to their different chemical/biochemical reactive moieties. Research devoted to the chemistry and physiology of BAs dates back to the beginning of the 20th century. Since then, it remains relevant to medicine and pharmacology thanks to the potential of several BAs as therapeutic agents. Primary and secondary BAs are the major components of bile; the former is sourced from cholesterol catabolism in the liver, while the latter is derived from the biotransformation of primary BAs by the action of intestinal microbiota [5,6]. BAs play various and complex key roles in health and disease, ensuring the intestinal solubilization and absorption of lipids, cholesterol, fat-soluble vitamins and hydrophobic drugs [7,8,9]. Apart from their conventional role as bio-surfactants, BAs are also considered hormones acting as signaling molecules, notably interacting with two receptors: the G-protein-coupled bile acid receptor-1 (GPBAR-1, also known as TGR5), and farnesoid X nuclear receptors (FXR) [7,10]. In this way, BAs regulate many physiological functions including lipid, glucose and energy metabolism, modulation of inflammatory and immunomodulatory processes [11,12,13,14]. As is well known, the biological functions of BAs are related to the physical-chemical properties mainly arising from the chemical structures of their amphiphilic molecules, i.e., with a concave hydrophilic α-face and a convex hydrophobic β-face (Figure 1A). The general hydrophobicity scale of BAs is as follows: ursodeoxycholic acid (UDCA) < cholic acid (CA) < chenodeoxycholic acid (CDCA) < deoxycholic acid (DCA) < lithocholic acid (LCA) (Figure 1B). Certain hydrophobic BAs have been shown to be cytotoxic and have been associated with cancer in several gastrointestinal organs including esophagus, stomach, small intestine, liver, biliary tract, pancreas and colon/rectum [15]. Thus, a complex series of molecular mechanisms related to the promotion of cancerogenesis by BAs, including oxidative stress with DNA damage, apoptosis, epigenetic factors regulating gene expression, activation or inactivation of nuclear receptors, alteration of gut microbiota, has been identified in recent decades [16]. On the other hand, recent studies have suggested that some hydrophilic BAs exhibit beneficial effects as anticancer agents as well, while modulating the same pathways which induce toxicity. In particular, UDCA and TUDCA (that is, the taurine conjugated of UDCA, Figure 1C)) show cytoprotective and chemopreventive effects, and UDCA exhibits weak to moderate anticancer activity in vitro when used at high concentrations [17].

Due to their peculiar physical-chemical and biological properties, together with the ease of carrying out chemical modification of hydroxyl and carboxyl groups, BAs have gained significant attention with respect to their use for the molecular hybridization of natural/synthetic and drug/nondrug scaffolds to design new compounds to improve the bioavailability [18], metabolic stability and intestinal absorption of parent compounds [19,20]. Moreover, the well-established ability of bile acids to regulate the activation or inhibition of several nuclear and transmembrane receptors (P-glycoprotein) has led to the development of novel bile acid conjugates to target these receptors that play a significant role in carcinogenesis and inflammation [21,22].

In this short review, we focus on BA hybrids with potential anticancer activity reported in literature in the last ten years, and we share some unpublished results from our research group.

## 2. Synthetic Pathways to Bile Acid Hybrid Molecules

**Figure 2:**
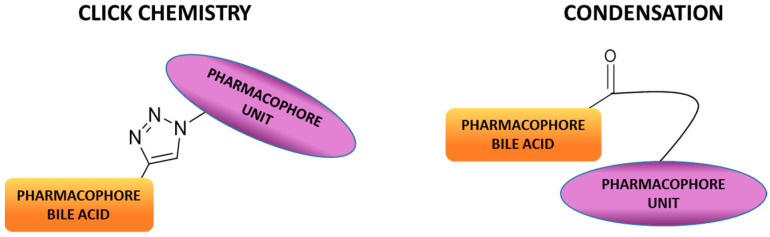
Sketch of BA-hybrids via click chemistry or condensation reaction.

The combination of two or more compounds into one covalently linked compound represents one option for the development of hybrid drugs. Such a connection can be accomplished through cleavable or no cleavable linkages, and the molecular entities can be linked directly or via a linking arm (Figure 2). The click reaction (the copper-catalyzed azide-alkyne cycloaddition Cu-AAC) is a very appealing approach to connect two different molecules using a no cleavable linkage [23]. The click is a highly specific and high yield reaction, and the resulting 1,2,3-triazole moiety can improve the pharmacological, pharmacokinetic and physiochemical profiles of bioactive compounds [24]. Indeed, the 1,2,3-triazole scaffold is frequently used in medicinal chemistry to replace amide and ester bonds because of its in vivo stability to both enzymatic and chemical degradation, and its ability to effect hydrogen bonding [25]. All these properties have resulted in a widespread occurrence of 1,2,3-triazole derivatives in different pharmacological active compounds such as fungicidal, antibacterial, anti-inflammatory, analgesic, antiviral, antitumoral, immunomodulatory, antimalarial, etc. Broad and efficacious activity has established triazoles and their derivatives as pharmacologically significant scaffolds [26,27]. On the other hand, the approach using cleavable bonds is based on the release of two molecular entities under enzymatic or physiological conditions. Condensation reactions are a simple and efficient method to achieve BA conjugates under basic or acidic conditions. For instance, bile acid C-24 position can be easily reacted with amino or alcohol moieties, yielding amide or ester derivatives. Amide and ester linkages are the most prevalent cleavable chemical bonds used to design and synthetize hybrid compounds. In fact, amide and ester bonds exist widely in many organic molecules and biomolecules because of their good stability (amide bonds in particular) under various conditions. Nevertheless, they can be hydrolyzed in the presence of amidases or esterases to release molecular entities.

## 3. Bile Acid Hybrid Molecules Based on Natural Bioactive Molecules

In the following section, we report selected examples of BAs conjugated with natural product derivatives including endogenous nucleosides and natural products first isolated form plants such as Dihydroartemisinin (DHA) and Camptothecin (CPT) which themselves demonstrate anticancer activity. Table 1 briefly summarizes BA hybrids based on natural pharmacophores, as well as improvements in biological activities achieved through conjugations.

**Scheme 1:**
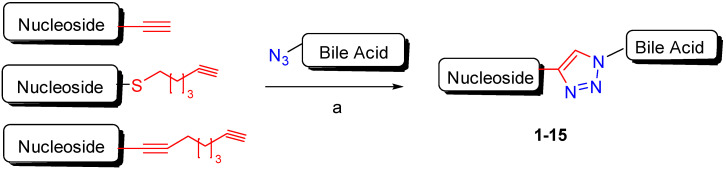
Synthetic approach to 1,2,3-triazole linked bile acid-nucleoside hybrids. Reaction conditions: (a) CuSO_4_·5H_2_O, THF:*t*BuOH:H_2_O (1.5:1:1), 25 °C, 18 h or MW 80 °C, 30 min. (68–85% yield).

The anticancer profiles of bile acid-nucleoside hybrids were reported in the last decade. The 1,2,3-triazole scaffold was chosen to connect bile acids to deoxyadenosine-nucleoside analogues via click chemistry and the corresponding hybrids were evaluated for their cytotoxicity against a panel of four different human cancer cell lines and normal fibroblast cells (Scheme 1) [28].

**Figure 3:**
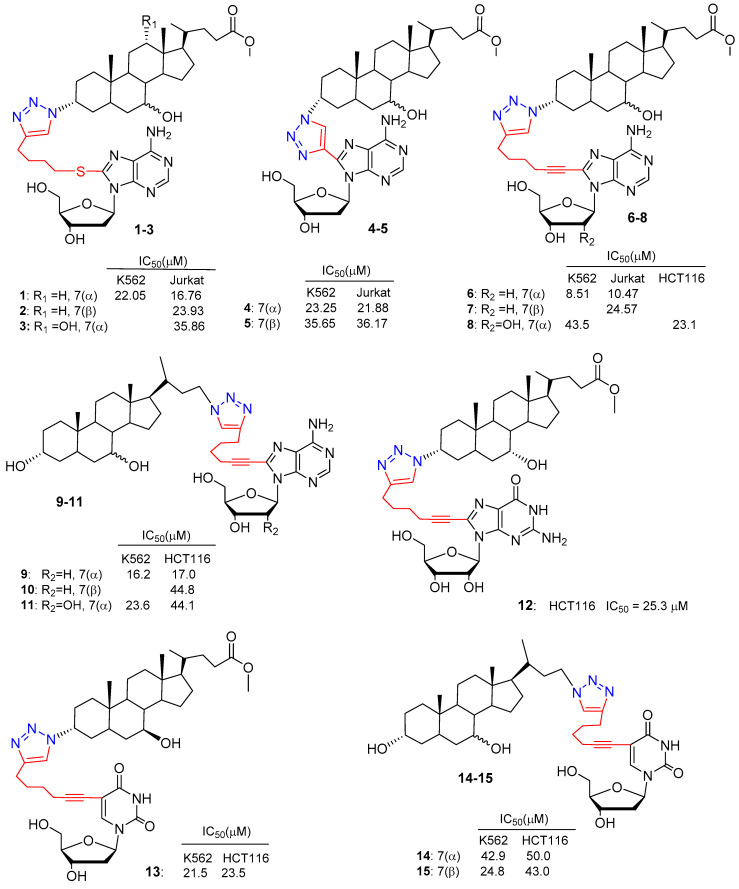
Cytotoxic activity of several BA-nucleoside hybrids linked via 1,2,3-triazole moiety.

A series of deoxyadenosines with three different alkynylic substituents on the C-8 position and the 3α-azido derivatives of cholic (C-N_3_), chenodeoxycholic (CDC-N_3_) and ursodeoxycholic acid (UDC-N_3_) were selected for the click conjugation (Scheme 1). Isolated yields of hybrid compounds were in the range 68–85% after chromatographic purification. The cytotoxic activity of conjugated compounds **1**–**7** (Figure 3) was evaluated against human cells of different histopathological origin: two leukemic cell lines (Jurkat and K562), a colon cancer cell line (HCT 116) an ovarian cancer cell line (A2780) and finally, normal human skin fibroblast cells as a control. Several conjugates incorporating the CDCA scaffold exhibited good concentration-dependent antiproliferative activity against both leukemia cell lines. In particular, compound **6** (CDC-deoxyadenosine derivative), the most potent, demonstrated cytotoxicity comparable to that of the positive control, Cisplatin, towards K562 and Jurkat cells with IC_50_ values of 8.51 and 10.47 respectively, whereas **6** did not affect normal fibroblast cell viability. UDC-based hybrids were less cytotoxic in general compared to CDC-based compounds, and cytotoxicity dramatically decreased with CA-based hybrids. No hybrid compounds showed cytotoxicity against HCT116, A2780 cell lines up to 200 µM concentration. Finally, C-8 alkynylated deoxyadenosine moieties seemed to play a crucial role in the selectivity of the cytotoxic process. Indeed, bile acid-1,2,3-triazole derivatives lacking deoxyadenosine scaffolds caused cell death in quite similar concentrations in all cell lines tested, including normal fibroblast cells.

More recently, a study on the synthesis and biological evaluation of a library of BA-nucleoside hybrids was reported (Figure 3, compounds **8**–**15**) [29]. All hybrid compounds were prepared via click chemistry under the optimized conditions previously reported (Scheme 1). All BA-based hybrids were evaluated in vitro for their cytotoxic activity against leukemic K562 cells and the colon cancer HCT116 cell line, as well as on normal human skin fibroblast. The results revealed that some compounds displayed interesting antiproliferative activity, with IC_50_ values ≤ 25 µM. Among them, CDC derivative **9** was the most active BA-based hybrid against both K562 and HCT116 cell lines, with IC_50_ values of 16.2 and 17.0 respectively. Moreover, it showed a high IC_50_ value (around 100 µM) toward normal fibroblast cells and induced apoptosis of leukemic K562 cells. The reported in vitro screening highlighted that: (i) CDC-scaffolds displayed a fair degree of cytotoxic activity and cytoselectivity when conjugated with adenosine/deoxyadenosine nucleoside derivatives; (ii) UDC-scaffolds displayed a moderate degree of cytotoxicity only when coupled with a pyrimidine nucleoside derivative (2′-deoxyuridine); (iii) in the case of TUDCA-based hybrids, as expected, no cytotoxic activity was observed. The overall data suggest that cytoselectivity is mainly driven by the nature of BA and can be tuned by the nature of the nucleoside derivative.

**Scheme 2:**
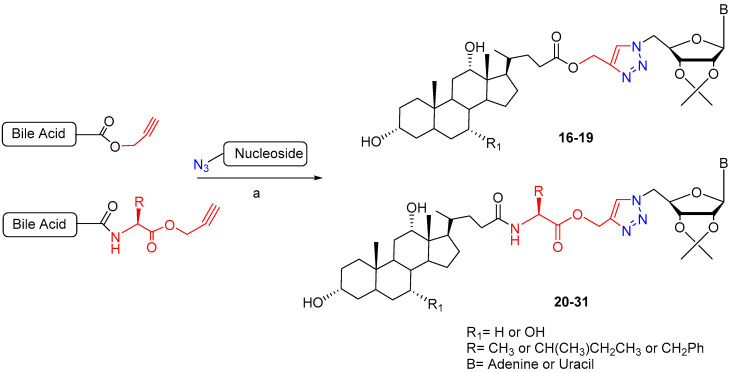
Synthesis of BA-nucleoside conjugates linked via triazolium ring. Reaction conditions: (a) CuSO_4_·5H_2_O, Na-ascorbate, DMF/H_2_O, rt, 1–6 h, 85–92% yield.

Another study reported the synthesis of a series of bile acid-nucleoside conjugates linked via a triazolium ring [30]. Two different types of bile acid ‘clicked’ nucleosides were prepared either linked directly or by amino acids (Scheme 2). To prepare directly clicked bile acid-nucleoside hybrids, CA and deoxycholic acid (DCA) were derivatized as propargyl esters. Meanwhile, to prepare amino acid-linked clicked bile acid-nucleosides, BAs were previously coupled with the selected amino acid propargyl ester. The 1,3-dipolar cycle addition of propargyl ester with 5′-N_3_-nucleosides was carried out using copper catalyzed conditions in DMF/water, affording conjugated compounds with excellent yields (85–92%).

The synthesized conjugates were screened against a panel of three cancer cell lines: PC-3 (human prostate cancer cell line), MCF-7 (human breast adenocarcinoma cell line), IMR-32 (human neuroblastoma cell line) and healthy HEK 293T (Human Embryonic Kidney) (Table 2).

Research on BA-nucleosides conjugates is limited to in vitro activity against a variety of cancer cells. Unless the conjugate molecules herein reviewed displayed cytotoxic activity in the micromolar range and some cytoselectivity, no advanced studies on their biological mechanisms or ex/in vivo experiments have been reported yet.

In recent decades, many studies have proven the potential anticancer activity of Artemisinin (ART), a secondary metabolite of *Artemisia annua*, a plant of the *Asteraceae* family of Chinese origin, with excellent properties against the *Plasmodium* parasites responsible for malaria, and its metabolites [37]. ART and ART derivatives are the gold standard for malaria treatment; however, their poor solubility and stability prevent their clinical use in chemotherapy despite their promising anticancer activity. Among ART metabolites, Dihydroartemisinin (DHA), the reduction product of ART, is characterized by a hemiacetal moiety at the C-10 position that improves water solubility with respect to the parent ART and offers the chance to undertake further chemical modifications.

**Scheme 3:**
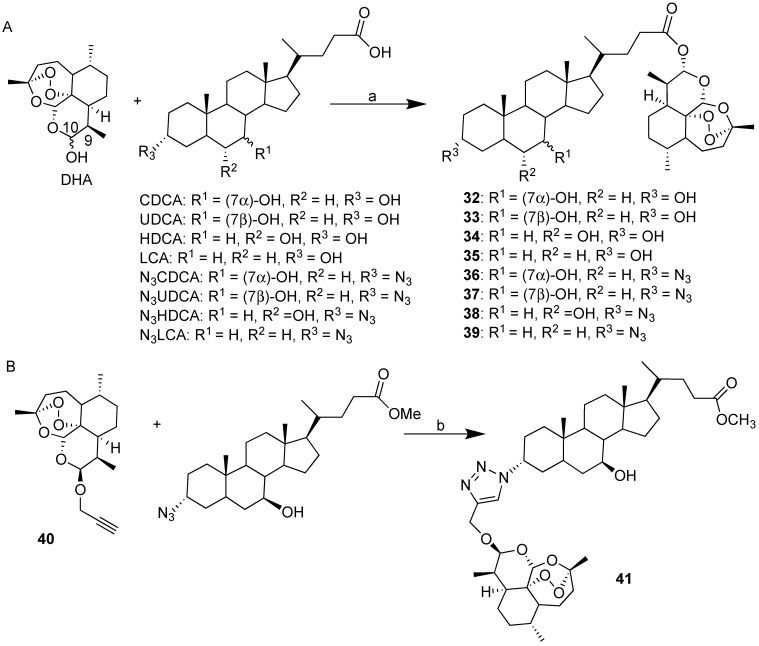
(**A**) Synthesis of BA-DHA hybrids trough condensation reaction. Reaction conditions: (a) EDCI, DMAP, DMF, 25 °C, 18 h, 25–60% yield; (**B**) Synthesis of UDC-DHA hybrid **41** via click chemistry. Reaction conditions: (b) propargyl-DHA/N_3_UDCMe ratio: 1/1, CuI 0.1 eq, room temperature, 18 h, 28% yield.

Recently, our research group explored convergent synthetic approaches to BA-DHA hybrids through both condensation reactions and click chemistry (Scheme 3) [31]. A simple condensation reaction mediated by EDCI between the appropriate bile acid (CDCA, UDCA, HDCA and LCA) and DHA revealed a successful synthetic strategy, yielding the corresponding BA-DHA hybrids in a satisfactory manner. The present synthetic strategy, being a one pot reaction, is very attractive, since all starting materials are commercially available. Following the same procedure, N_3_CDC-DHA and N_3_UDC-DHA hybrids starting from the corresponding 3-azido derivatives of CDCA and UDCA were also prepared (Scheme 3A). BA-DHA hybrids obtained by condensation reaction (Scheme 3B) are linked through a biologically labile ester bond; therefore, they are expected to act as prodrugs. To obtain BA-DHA hybrids that are more stable under physiological conditions, in the case of UDCA, click chemistry was explored (Scheme 3B). Propargyl-DHA **40** [38] was reacted in a 1:1 ratio with N_3_UDCMe [39] in the presence of CuI in acetonitrile at room temperature (Scheme 3B)

Hybrid **32** (CDC based) was preliminarily tested in vitro against a selection of Diffuse Large B Cell Lymphoma (DLBCL) such as RAJI, U2932, SUDHL-4, KARPAS-422 and BL-2. Lymphoma is the most common blood cancer that can affect both T lymphocytes (T-cells) and B lymphocytes (B-cells). T-cell non-Hodgkin lymphomas are relatively uncommon, accounting for around 15% of all lymphomas, but are associated with a poor clinical outcome [40]. DLBCL is one of the most common types of aggressive B-cell non-Hodgkin lymphoma that can grow in lymph nodes as well as outside of the lymphatic system, infiltrating tissues and obstructing organs. The large number of patients who are refractory to treatments or relapse afterwards strongly encourages the development of new anticancer drugs [41].

**Figure 4:**
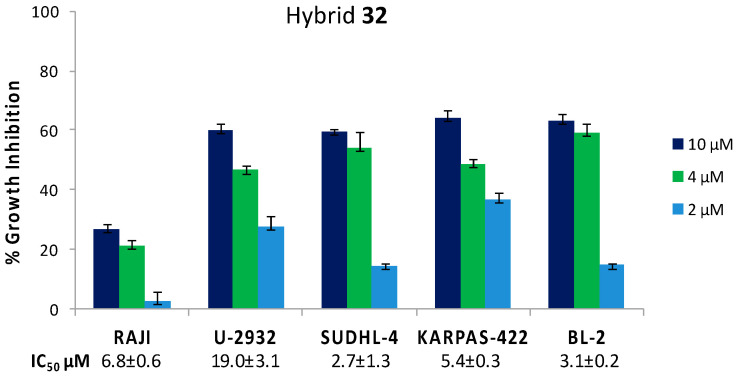
Antiproliferative activity of hybrid **32** against a selection of B-lymphoma cells at 10, 4, 2 µM concentration at 72 h incubation time. IC_50_ values are expressed as the mean of three independent experiments ± SD.

Cell growth inhibition was evaluated at concentrations of 10, 4 and 2 µM after 72 h incubation time. Hybrid **32** showed a significant concentration-dependent antiproliferative effect against all the tested DLBCL. Apart from the RAJI cell line, for which an IC_50_ value of around 20 µM was extrapolated, single digit IC_50_ values in the low micromolar range were found (Figure 4) [32].

**Figure 5:**
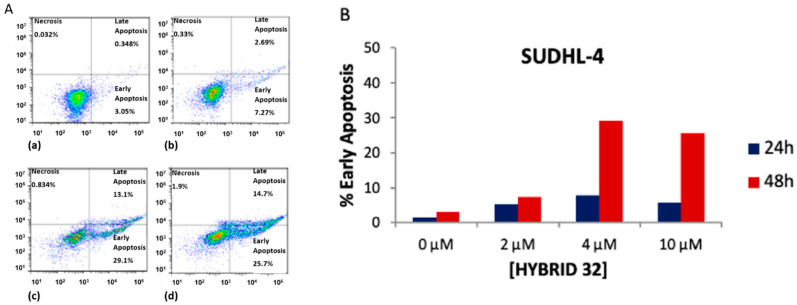
(**A**) Hybrid **32** induced apoptosis in SUDHL-4 cells after 48 h treatment: (a) control: untreated cells; (b) **32**, 2 µM; (c) **32**, 4 µM; (d) **32**, 10 µM. (**B**) % early apoptosis in function of **32** concentrations at different treatment times.

Furthermore, the mechanism of cell death was investigated in order to evaluate the proapoptotic effect of **32** in the most sensitive SUDHL-4, KARPAS-422 and BL-2 cancer cell lines. The selected DLBCL cells were treated with **32** (2, 4 and 10 µM) for 24 and 48 h, and then assayed by flow cytometry analysis with Annexin V-FITC staining. As depicted in Figure 5, a highly predominant dose-/time-dependent apoptotic mechanism of antiproliferative activity with high early apoptosis percentage was found in SUDHL-4. Similar results were obtained in the KARPAS-422 and BL-2 cells [32].

Artemether (C10-OCH_3_ dihydroartemisinin derivative) has been shown to inhibit proliferation in SUDHL-4, leading to 70% growth inhibition at 100 µM concentration after 72 h incubation time [42]. In the case of hybrid **32**, we found a similar percentage of growth inhibition at 10 times lower concentration (ca. 10 µM) (Figure 5), thereby confirming that hybridization can significantly improve the cytotoxic activity in cancer cells.

The remarkable anticancer activity of DHA towards leukemia and hepatocellular carcinoma (HCC) was recently revealed. HCC is the fourth most common cause of cancer-related death worldwide. Surgery and transplantation can be effective treatments in early-stage HCC, whereas drug therapy can improve the prognosis by slowing the progression of the disease in people with advanced liver cancers. DHA is known to significantly inhibit HCC cell growth in vitro and in vivo, but its poor stability limits its utilization [43,44,45,46]. Therefore, DHA conjugation with BA which is able to target hepatocyte transporters may be desirable. The BA-DHA hybrids reported in Scheme 3 were tested against HCC HepG2 [31] and Huh-7 [33], as well as epithelial healthy cells [31]. Cytotoxicity was evaluated using a MTT assay. A negligible cytotoxic effect was found toward epithelial normal cells with IC_50_ values > 100 μM in all cases. In turn, the results summarized in Table 3 show enhanced cytotoxic activity for all hybrids (except for compound **36** N_3_-CDC based) with respect to DHA alone toward both HepG2 and Huh-7 cancer cells. Remarkable improvement was found in the cases of **33**, **34**, **37** and click hybrid **41** (UDC based), for which conjugation improved HepG2 growth inhibition ≥ 10 fold. Huh-7 cells were found to be less sensitive than HepG2 with respect to DHA with IC_50_ of 39.96 μM compared to 21.3 μM in HepG2. However, in the case of hybrid **33**, comparable cytotoxic activity was found in both the HCC HepG2 and Huh-7 lines with IC_50_ of 2.16 μM and 1.75 μM respectively. The building blocks were also tested in both HepG2 and Huh-7 cells, but the cytotoxicity was very low, with IC_50_ values > 100 μM. In the case of leukemia HL-60, all hybrids showed enhanced cytotoxicity with respect to DHA alone; in particular, hybrids **33** and **37** showed 10.5- and 8.3-fold increases in growth inhibition, respectively (Table 3). The effectiveness of conjugation on the stability of DHA compared to DHA alone was revealed by a HPLC/MS stability study in a cell culture medium of DHA, and the most promising hybrid, **33,** was found to be 100% stable after 24 h, whereas a t ½ of ca. 4 h was found for DHA [33].

**Scheme 4:**
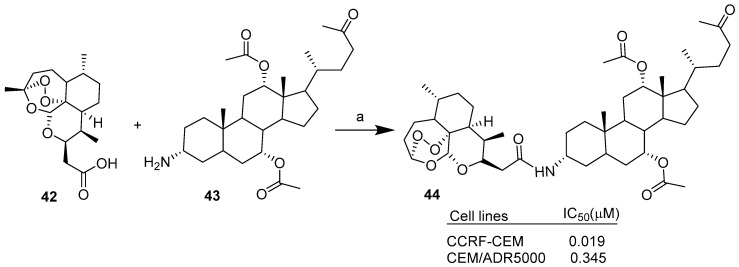
BA-DHA-derived hybrids. Reaction conditions: a, EDCI·HCl, DMAP, DCM, 24 h, 86% yield.

C-10 substituted dihydroartemisinin derivatives such as the artesunic acid, as well as deoxoartemisinin carboxylic acid and alcohol derivatives, were conjugated with cholic acid (CA) derivatives through condensation reactions mediated by EDCI [34]. Scheme 4 reports the synthesis of the most active hybrid as an example. The novel hybrids were tested on sensitive CCRF-CEM and multidrug-resistant CEM/ADR5000 leukemia cells. All hybrids showed improved cytotoxic activities against the selected leukemia cancer cell lines compared to the ART unit alone. Moreover, the combination of several semisynthetic DHA derivatives with several CA derivatives provided insights into the structure–activity relationships. In particular, hybrid **44** was found to be the most potent compound against both CCRF-CEM and CEM/ADR5000 cells, with IC_50_ values of 0.019 µM and 0.345 µM, respectively. In the case of CEM/ADR5000 multidrug-resistant cancer cells, hybrid **44** showed better cytotoxicity than Doxorubicin (IC_50_ = 1.61 µM).

This work demonstrated that the hybridization of semisynthetic artemisinins derivatives with appropriate CA derivatives can lead to novel hybrids with improved biological activities respect the parent ART. The structure–activity relationship study also highlighted some key points that, together, enhance the cytotoxic activity of target hybrid **44**: the α-orientation at C-3 position of the cholic acid moiety, the incorporation of a C-10 nonacetal artemisinin unit (deoxoartemisinin) and the linkage between them via a two-carbon chain.

Research on bile acid-artemisinins conjugates is very recent; all results herein reported were published in or after 2017, and no in vivo studies have been reported yet. However, many studies have been published on the use of artemisinins as anticancer agents in combination with chemotherapeutics (i.e Cytarabine, Cisplatin, Gemcitabine etc.) or phytochemicals, indicating that that the combination approach involving artemisinins can improve anticancer activity and lead to new drug partners for clinical application. Some clinical trials involving the treatment of advanced or metastasized solid tumors with artemisinins have been also reported, encouraging further study of artemisinins conjugates [37].

Camptothecin (CPT), a natural alkaloid first isolated form the Chinese tree *Camptotheca acuminata*, presents potent anticancer activity through the inhibition of topoisomerase I against leukemia and several solid tumors including colon and hepatic cancer. The negligible water solubility and poor stability at the lactone ring of CPT limit its clinical use and strongly support a prodrug strategy [47].

**Scheme 5:**
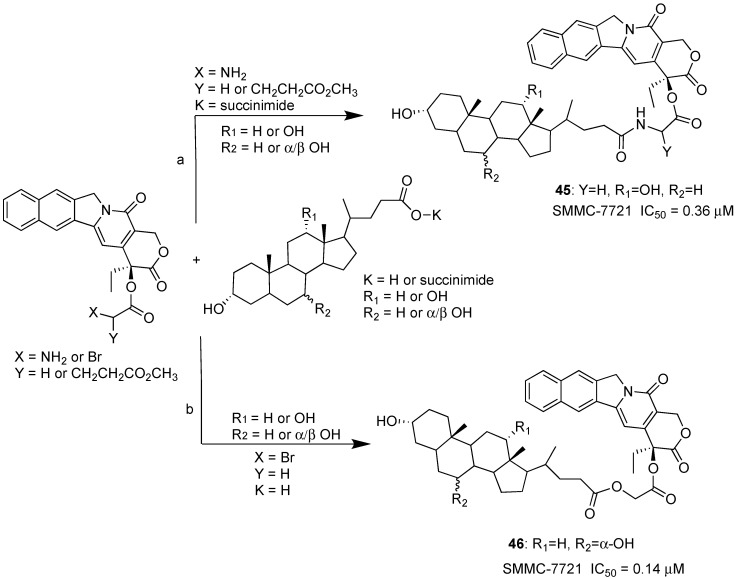
Synthesis of BA-CPT hybrids. Reaction conditions: (a) TEA and DMSO; (b) TEA and DMF; 65–75% yield.

Recently, the synthesis was reported of a series of BA-CPT hybrids obtained by condensation reaction of CPT, modified with a suitable linker, and CA, DCA, UDCA and CDCA or their succinimide C-24 derivative (Scheme 5) [35]. All BA-CPT hybrids were tested against a selection of solid tumors such as MCF-7 breast, HCT-116 colon and SMMC-7721 liver cancer. The best cytotoxic activity was found against the SMMC-7721 hepatoma cell line. In particular, hybrids **45** and **46** showed better inhibition than CPT alone (IC_50_ = 0.37, 0.14 and 0.56 μM respectively). Stability studies in blood rat serum of hybrid **45** showed that conjugation actually stabilizes CPT, resulting in an increase of hybrid half-life to up to 24 h, compared to a 2.3-h half-life of CPT alone. More recently, the same authors demonstrated in vitro and in vivo that DC-CPT hybrid **46** was more stable than CTP alone, and that its potential to target liver was remarkably enhanced by deoxycholic bile acid conjugation [36].

## 4. Bile Acid Hybrid Molecules Based on Synthetic Bioactive Molecules

In the following section, we report selected examples of BA conjugated with synthetic drugs such as Tamoxifen (TAM) and Cytarabine (Ara-C), as well as functional group or aromatic bioactive synthetic units. Table 4 briefly summarizes BA hybrids based on synthetic pharmacophores and improvements in biological activities achieved through conjugation.

**Scheme 6:**
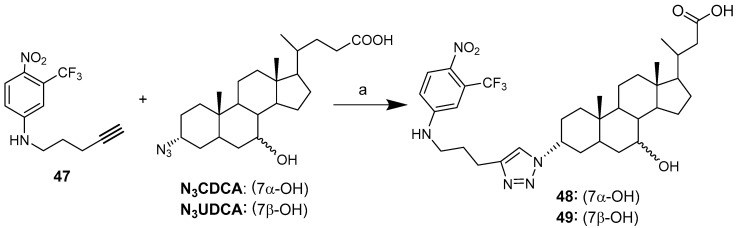
Reaction conditions: (a) CuSO_4_·5H_2_O, sodium ascorbate, THF:*t*-BuOH:H_2_O (1.5:1:1), microwaves: 80 °C, 30 min; 70–80% yield.

Upon irradiation with visible light, 1-nitro-2-(trifluoromethyl)aniline alkyne, a nitic oxide (NO) photodonor molecule, is able to generate NO radicals [48]. NO can be used as an anticancer agent or as a chemosensitizer to enhance the effect of traditional cancer therapies [53,54]. Bile acid UDCA and CDCA 3-azido derivatives were conjugated with 1-nitro-2-(trifluoromethyl)aniline alkyne **47** by click chemistry (Scheme 6) [48]. This original feature provides the opportunity to combine the intrinsic characteristics of biological components with those of NO radicals, yielding interesting tools for cancer therapy and representing, in principle, a relevant example of hybrids with a dual mode of action.

Both hybrids showed a significant concentration-dependent antiproliferative effect towards both K562 and HCT116 cancer cells lines. Hybrid **49** (UDC based) displayed greater cytoselectivity than **48** (CDC based), with the latter exhibiting comparable cytotoxity toward cancer and normal cells. (Table 5).

The effective release of NO from photoactive conjugates **49** was assessed by irradiation with visible light of the conjugate in solution. NO release was measured by means of an ultrasensitive NO electrode by an amperometric technique. This experiment demonstrated the preservation of the photobehavior of the photoactive center after its covalent linkage with bile acid UDC. The success of photochemical studies on the effective release of NO have led hybrid **49** to be considered as a progenitor of a large variety of new bioconjugates combining the intrinsic characteristics of biological components with those of NO, giving rise to potentially powerful tools in cancer therapy.

Tamoxifen (TAM) belongs to the family of nonsteroidal triphenylethylene derivatives, and is a selective estrogen receptor modulator. Tamoxifen has been widely used as a treatment for breast cancer since the 1970s. Despite its widespread use, several drawbacks, such as high hepatic and intestinal first pass metabolism, hepatotoxicity, together with poor oral bioavailability, have prompted the search for drug conjugates that can help cellular penetration, leading to enhanced therapeutic activity and lower toxicity. In this regard, the synthesis of a series of BA-TAM hybrids bearing acid and amine heads, and their anticancer activities and mechanisms of action in ER +ve and ER −ve breast cancer cells were recently reported [49].

**Scheme 7:**
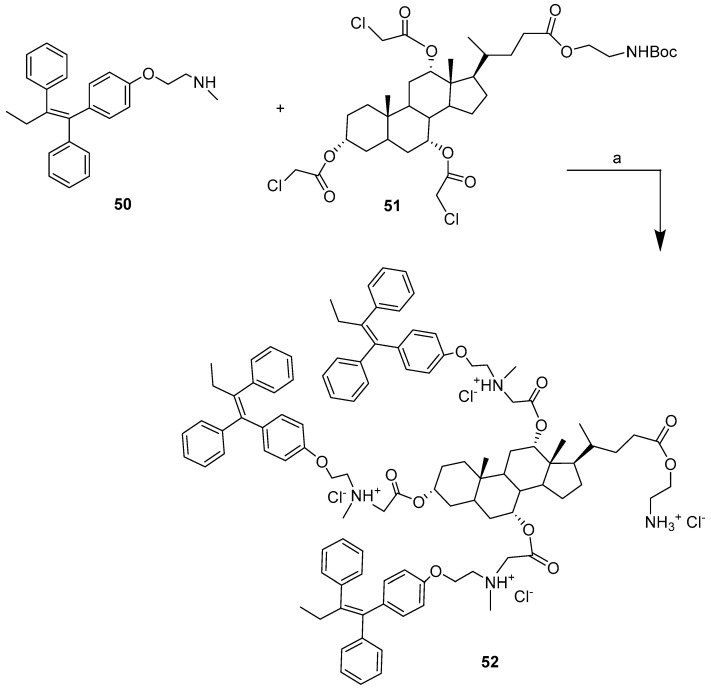
Synthesis of CA-TAM hybrid. Reaction conditions: (a) DCM, TFA, 6 h, r.t.

Bile Acid−TAM conjugate **52** (cholic acid and three unit of TAM), prepared by condensation reaction between TAM derivative **50** and cholic bile acid derivative **51** (Scheme 7), was found to be the most active hybrid compared to TAM alone in three out of four cancer cell lines, with at least a two-fold increase in activity (Table 6).

Hybrid **52** was tested in vivo for anticancer activity in the murine 4T1 breast cancer model in Balb/c mice. Also, in vivo hybrid **52** was found to be more potent compared to TAM alone, causing a ∼50% reduction in tumor volume with a single dose, whereas a single dose of TAM was not effective at all.

**Scheme 8:**
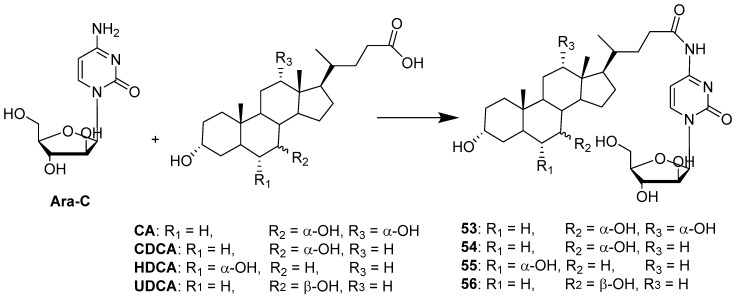
Synthesis of BA-Ara.C conjugates. Reaction conditions: Ara-C:BA:TEA:ClCOOCH_2_(CH_3_)_2_ (1:1.2:1.5:1.); −15 °C; 30 min; 78–87% yield.

Cytarabine (Ara-C), a deoxycytidine analogue, is one of the most effective drugs for the treatment of acute myeloid leukemia. It is also used to treat Hodgkin’s lymphoma, as well as meningeal leukemia and other types of lymphoma [55]. On the other hand, the use of cytarabine in the treatment of solid tumors is limited. Ara-C itself is quite a powerful drug, but there are some shortcomings limiting its use and efficiency, such as low lipophilicity and low stability leading to rapid enzymatic deamination. Therefore, the conjugation of Ara-C is an interesting approach to enhance lipophilicity and stability, as well as to expand its biological space. The choice of BA as conjugation partners could accomplish many goals: a simple condensation reaction between the 4-amino group of cytarabine (4-NH_2_) and the carboxylic group of BA at C-24 position (Figure 1) yields a stable amide; BA covalently bound to Ara-C improves lipophilicity; BA expresses specific transporters in enterocytes and hepatocytes, and therefore, BA conjugation can expand the range of biological activity of Ara-C toward solid tumors such as hepatocarcinoma. Sun et al. [50] reported the synthesis, through condensation reaction, and a biological evaluation of four conjugates of Ara-C with CA, CDCA, HDCA and UDCA (**53**–**56**) (Scheme 8).

All conjugates tested on HepG2 cancer cells showed increased antiproliferative activity as a result of the increased drug bioavailability via AOTP-mediated transport. Moreover, a pharmacokinetic study in rats showed that Ara-C conjugated with UDCA increased the oral availability of Ara-C by two fold. The overall data indicated that Ara-C conjugation with a bile acid may be a successful strategy to improve the stability and oral absorption of Ara-C.

**Scheme 9:**
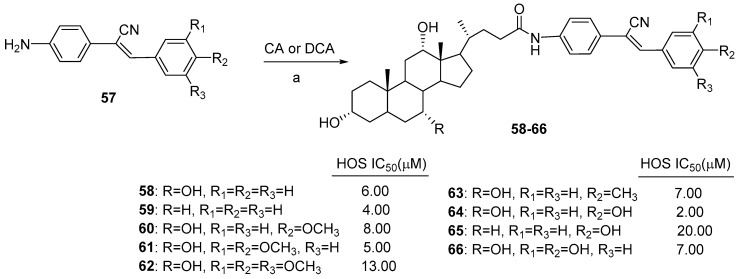
Synthesis of α-cyanostilbene hybrids. Reaction condictions: (a) EDC, HCl/HOBt/Et_3_N, DMF, MW, 80 °C, 1 h (52–88% yields).

Cholic and deoxycholic acid amides were conjugated with a series of amino-substituted α-cyanostilbene derivatives, and their anticancer activity was evaluated against the human osteosarcoma (HOS) cancer cell line [51]. α-Cyanostilbenes are usually considered as resveratrol analogs, displaying good anticancer activity against a range of cancer cell line [56]. For the synthesis of hybrid compounds, α-cyanostilbenes were prepared with 80–85% overall yields by Knoevenagel condensation between 2-(4-nitrophenyl) acetonitrile and various aldehydes with catalityc amounts of piperidine, followed by reduction of the nitro group (Scheme 9). The coupling of CA (or DCA) with amine-substituted α-cyanostilbenes **57** was carried out with using EDC·HCl/HOBt/NEt_3_ in DMF, yielding cholic acid (or deoxycholic acid) amides of α-cyanostilbenes **58**–**66** in 52–88% yields (Scheme 9). All the synthesized cholic acid-based hybrids displayed IC_50_ values in the scale of 2–13 μM against HOS; **64** was the most active compound (IC_50_ = 2 μM). In the case of deoxycholic-based α-cyanostilbene derivatives, only two hybrids (**59** e **65**) were found to be active. Moreover, an in vitro test showed that cholic acid α-cyanostilbene conjugates having one or more strong electron-donating group such as OH or OCH_3_ were more active than their corresponding α-cyanostilbene derivatives. An in vitro cell cycle analysis of compound **64** indicated that it arrests cell growth in the G1 phase and induces apoptosis.

**Scheme 10:**
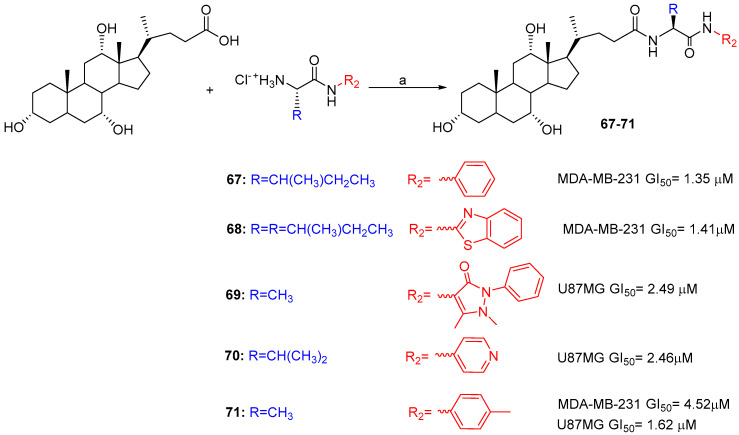
Synthesis of aryl/heteroaryl hybrids. Reaction conditions: (a) EDC·HCl/HOBt/DMF, r.t. 6–8 h.

Another study reported on the cytotoxicity of BA-based aryl/heteroaryl hybrids linked through α-amino acids [52]. The synthetic strategy involved the coupling of L-aminoacyl aromatic and heteroaromatic amides with CA and DCA using EDC·HCl/HOBt in DMF at room temperature (Scheme 10). All conjugated compounds were evaluated to investigate their antiproliferative activity against three different human cancer lines, i.e., colon, breast and glioblastoma cancers (HT-29, MDA-MB-231 and U87), and cytotoxicity on a normal cell line (HEK293T). Several cholic acid derivatives (**67**, **68** and **71**) showed good activity against the breast cancer cell line (GI_50_ = 1.35, 1.41 and 4.52 μM respectively) compared to Cisplatin (7.21 μM) and activity comparable to Doxorubicin (1 μM), while **69**, **70** and **71**, showed better activity against the glioblastoma cancer cell line (GI_50_ = 2.49, 2.46 and 1.62 μM respectively) compared to both Cisplatin (3.78 μM) and Doxorubicin (2.60 μM), drugs which are routinely used in the treatment of this form of cancer. More than 65% of the compounds were found to be safe on a human normal cell line. Based on literature reports, a plausible mode of mechanism has been inferred for cytotoxic conjugates.

## 5. Conclusions

Combination therapy approaches can help to overcome drug resistance as well as to reduce toxicity. While there is considerable literature on the use of pharmacophores in combination with chemotherapeutics or phytochemicals, pharmacophore hybridization, that involves the conjugation of at least one bioactive molecule through a covalent bond, is a more recent approach. The current review reported recent developments in the chemistry and biology of a particular group bioconjugates based on bile acid derivatives in the hope of motivating the further development of this family of molecules. Some of the new bile acid-based hybrids herein described include widely used anticancer drugs, for instance Cytarabine and Tamoxifen, that require conjugation to improve their properties and expand their biological space. Regarding natural artemisinin derivatives, conjugation is necessary for the development of novel anticancer drugs, since the poor solubility, poor stability and toxicity of artemisinins prevent their clinical use in chemotherapy despite their well assessed anticancer activity.

It is well known that BA can enhance the bioavailability, oral adsorption, cellular selectivity and site-specific delivery of conjugated compounds, thanks to their amphiphilic properties, stability to dynamic pH variations, as well as their ability to target specific receptors. However, additional efforts are required to better understand the molecular mechanisms of bile acid-based drugs in biological systems in order to potentially implement them in clinical settings and design new and more potent derivatives with activity against tumor growth.

Finally, in recent years, BA have drawn attention in the field of drug delivery also for their ability to act as drug carrier systems in the form of mixed micelles and bilosomes. The peculiar characteristics of BA acid to form liposomes encourages BA drug hybrids for in vivo studies through oral or intravenous delivery.

## Figures and Tables

**Table 1:** Summary of BA hybrids.

Hybrid	Conjugation Type	Cancer Cell Line	Mechanism/Event	Ref.
BA-nucleosides	Click	K562, Jurkat, HCT-116, MCF-7, IMR-32.	Induce apoptosis, better selectivity than Cisplatin.	[28,29,30]
BA-DHA	Click/Condensation	K562, RAJI, U-2932, SUDHL-4, KARPAS-422, BL-2, HL-60, HepG2, Huh-7.	Improved cytotoxicity, induce apoptosis and G0/G1 arrest, ROS.	[31,32,33]
	Condensation	CCRF-CEM, CEM/ADR5000.	Improved cytotoxicity, comparable/better cytotoxicity than Doxorubicin.	[34]
BA-CPT	Condensation	MCF-7, HCT-116, SMMC-7721.	Better inhibition, enhanced stability.	[35,36]

**Table 2:** In vitro anticancer activity evaluation of **16**–**31** by MTT assay.

Compound			IC_50_ [µM]	
		PC-3	MCF-7	IMR-32
**16**	R_1_ = OH, B = A	16.35	17.06	17.45
**17**	R_1_ = H, B = A	13.50	16.76	16.06
**18**	R_1_ = OH, B = U	17.89	8.08	16.8
**19**	R_1_ = H, B = U	16.69	>100	16.68
**20**	R_1_ = OH, B = A, R = CH_3_	31.47	18.15	82.93
**21**	R_1_ = H, B = A, R = CH_3_	16.12	>100	16.01
**22**	R_1_ = OH, B = A, R = CH(CH_3_)CH_2_CH_3_	16.41	13.76	17.12
**23**	R_1_ = H, B = A, R = CH(CH_3_)CH_2_CH_3_	17.06	16.39	41.04
**24**	R_1_ = OH, B = A, R = CH_2_Ph	14.23	14.96	19.53
**25**	R_1_ = H, B = A, R = CH_2_Ph	17.63	20.09	14.42
**26**	R_1_ = OH, B = U, R = CH_3_	17.16	14.30	14.30
**27**	R_1_ = H, B = U, R = CH_3_	16.38	>100	31.69
**28**	R_1_ = OH, B = U, R = CH(CH_3_)CH_2_CH_3_	18.01	>100	18.23
**29**	R_1_ = H, B = U, R = CH(CH_3_)CH_2_CH_3_	>100	17.33	15.38
**30**	R_1_ = OH, B = U, R = CH_2_Ph	16.24	>100	8.71
**31**	R_1_ = H, B = U, R = CH_2_Ph	16.17	>100	18.59

**Table 3:** IC_50_ values were determined from the dose-response curves using MTT assay after 72 h incubation time. Results are expressed as the mean of three independent experiments ± SD. DHA/Hybrid ratio meant the ratio of the corresponding IC_50_ values.

	HepG2 [31]		Huh-7 [33]		HL-60 [31]	
	IC_50_ µM	DHA/Hybrid	IC_50_ µM	DHA/Hybrid	IC_50_ µM	DHA/Hybrid
DHA	21 ± 2	-	39.96 ± 1.31	-	2.0 ± 0.4	-
**32**	3.8 ± 0.4	5.5	14.41 ± 2.18	2.8	0.5 ± 0.06	5.5
**33**	1.8 ± 0.2	11.7	2.16 ± 0.39	18.5	0.19 ± 0.03	10.5
**34**	2.0 ± 0.2	10.5	6.03 ± 0.68	6.62	0.35 ± 0.06	5.7
**35**	5.7 ± 0.7	3.7	5.97 ± 0.74	6.69	0.41 ± 0.05	4.9
**36**	20 ± 2	1.1	50.30 ± 2.45	0.78	0.68 ± 0.01	2.9
**37**	2.2 ± 0.4	9.5	4.13 ± 0.84	9.68	0.24 ± 0.02	8.3
**38**	14.2 ± 0.7	1.5	15.92 ± 1.28	2.72	1.7 ± 0.2	1.2
**39**	4.7 ± 0.4	4.7	5.08 ± 0.44	8.18	0.35 ± 0.04	5.7
**41**	1.7 ± 0.2	12.4	-	-	0.328 ± 0.002	6.1

**Table 4:** Summary of BA hybrids.

Hybrid	Conjugation Type	Cancer Cell Line	Mechanism/Event	Ref.
BA-NO_donor_	Click	K562, HTC116.	Improved cytotoxicity and cytoselectivity.	[48]
BA-TAM	Condensation	4T1, MCF-7, T47D.	Better inhibition.	[49]
BA-Ara-C	Condensation	HepG2	Increased bioavailability via AOTP-mediated transport.	[50]
BA-α-Cyanostilbene	Condensation	HOS	Induce apoptosis and G1 arrest.	[51]
BA-Aryl/heteroaryl	Condensation	MDA-MB-231, U87.	Comparable/better cytotoxicity than Cisplatin and Doxorubicin	[52]

**Table 5:** IC50 values were determined from the dose−response curves using MTT assay after 72 h incubation time in the dark. Results are expressed as the mean of three independent experiments ± SD. Cisplatin was used as a reference compound.

	IC_50_ µM		
Compound	K562	HTC116	Fibroblast
**48**	24.0 ± 1.2	21.0 ± 0.8	32.0 ± 1.0
**49**	31.6 ± 1.6	14.0 ± 1.0	>>100
Cisplatin	5.40 ± 1.0	8.5 ± 1.2	25.4 ± 3.5

**Table 6:** IC_50_ (μM) Values of TAM and CA-TAM conjugate **52** with respect to TAM equivalent concentrations in different ER +ve and ER −ve cancer cell lines.

	IC_50_ µM			
Compound	4T1	MCF-7	T47D	MDA-MB 231
TAM	12.25 ± 1.92	18.25 ± 4.19	22.41 ± 2.15	19.41 ± 4.47
**52**	5.28 ± 4.32	8.1 ± 3.82	9.42 ± 4.21	17.55 ± 5.85
